# Localizatome: a database for stress-dependent subcellular localization changes in proteins

**DOI:** 10.1093/database/baaf028

**Published:** 2025-04-21

**Authors:** Takahide Matsushima, Yuki Naito, Tomoki Chiba, Ryota Kurimoto, Keiko Itano, Koji Ochiai, Koichi Takahashi, Naoki Goshima, Hiroshi Asahara

**Affiliations:** Department of Systems BioMedicine, Graduate School of Medical and Dental Sciences, Institute of Science Tokyo, 1-5-45 Yushima, Bunkyo-ku, Tokyo 113-8510, Japan; Department of Systems BioMedicine, Graduate School of Medical and Dental Sciences, Institute of Science Tokyo, 1-5-45 Yushima, Bunkyo-ku, Tokyo 113-8510, Japan; Department of Systems BioMedicine, Graduate School of Medical and Dental Sciences, Institute of Science Tokyo, 1-5-45 Yushima, Bunkyo-ku, Tokyo 113-8510, Japan; Department of Systems BioMedicine, Graduate School of Medical and Dental Sciences, Institute of Science Tokyo, 1-5-45 Yushima, Bunkyo-ku, Tokyo 113-8510, Japan; Department of Biomolecular Science and Engineering, SANKEN, Osaka University, 2-1 Yamadaoka, Suita, Osaka 565-0871, Japan; Laboratory for Biologically Inspired Computing, RIKEN Center for Biosystems Dynamics Research, 6-2-3 Furuedai, Suita, Osaka 565-0874, Japan; Laboratory for Biologically Inspired Computing, RIKEN Center for Biosystems Dynamics Research, 6-2-3 Furuedai, Suita, Osaka 565-0874, Japan; Molecular Profiling Research Center for Drug Discovery, National Institute of Advanced Industrial Science and Technology, 2-3-26 Aomi, Koto-ku, Tokyo 135-0064, Japan; Department of Human Science, Faculty of Human Science, Musashino University, 3-3-3 Ariake, Koto-ku, Tokyo 135-8181, Japan; Department of Systems BioMedicine, Graduate School of Medical and Dental Sciences, Institute of Science Tokyo, 1-5-45 Yushima, Bunkyo-ku, Tokyo 113-8510, Japan; Department of Molecular Medicine, Scripps Research, 10550 North Torrey Pines Road, MBB-102, La Jolla, CA 92037, United States

## Abstract

Understanding protein subcellular localization and its dynamic changes is crucial for elucidating cellular function and disease mechanisms, particularly under stress conditions, where protein localization changes can modulate cellular responses. Currently available databases provide insights into protein localization under steady-state conditions; however, stress-related dynamic localization changes remain poorly understood. Here, we present the Localizatome, a comprehensive database that captures stress-induced protein localization dynamics in living cells. Using an original high-throughput microscopy system and machine learning algorithms, we analysed the localization patterns of 10 287 fluorescent protein-fused human proteins in HeLa cells before and after exposure to oxidative stress. Our analysis revealed that 1910 proteins exhibited oxidative stress-dependent localization changes, particularly forming distinct foci. Among them, there were stress granule assembly factors and autophagy-related proteins, as well as components of various signalling pathways. Subsequent characterization identified some specific amino acid motifs and intrinsically disordered regions associated with stress-induced protein redistribution. The Localizatome provides open access to these data through a web-based interface, supporting a wide range of studies on cellular stress response and disease mechanisms.

Database URL

https://localizatome.embrys.jp/

## Introduction

The subcellular localization of proteins is important for understanding their function. Several databases already provide information on the subcellular localization of proteins in mammalian cells under steady-state conditions [1–3]; however, the localization of many proteins dynamically changes in response to various stresses. Therefore, we developed a system to analyse these stress-dependent changes in subcellular localization within the same cell and constructed a database known as ‘Localizatome (Localize-a-tome)’ to facilitate access to the data. To analyse these dynamic localization changes, we observed individual cells transiently expressing 10 287 fluorescent protein-fused human gene libraries using a customized high-throughput microscopy system, which included a plate transport robot. We analysed the resulting large-scale image data using a machine learning program that detects and scores stress-dependent localization changes, particularly protein accumulation, within the same live cells. The Localizatome database not only provides information on the steady-state subcellular localization of proteins, similar to existing databases, but also provides information on the dynamic localization changes that occur in response to stress, making it a valuable tool for stress-related research.

In this study, we focused on oxidative stress as a representative type of stress. Oxidative stress disrupts cellular homeostasis and is involved in various pathological conditions, including ageing and cancer [4]. It is caused by the excessive production of reactive oxygen species, including superoxide, hydrogen peroxide, singlet oxygen, ozone, hypohalous acids, and organic peroxides [5]. Although proteins under oxidative stress can undergo structural changes, misfolding, and accumulation [6], oxidative stress also triggers various regulatory mechanisms that enable proteins to undergo functional changes and modulate cellular stress responses through altered localization patterns. Understanding the behaviour of proteins under conditions of oxidative stress is important for elucidating the fundamental mechanisms of ageing, the various diseases involving oxidative stress, and the development of new treatment strategies. Numerous studies have comprehensively analysed the quantitative and qualitative changes that occur in proteins under oxidative stress [7, 8]. Some of these proteins, whose quantities and qualities change, also exhibit altered cellular localization. For example, stress granule assembly factors form a membrane-less functional organelle known as a stress granule, which regulates protein translation and other processes in an oxidative stress-dependent manner [9–11]. By accumulating information on the changes in cellular localization combined with existing next-generation sequencing and proteomics analysis, we provide further insight into the biological processes affected by oxidative stress.

In this study, we individually expressed 10 287 fluorescent protein-fused human proteins in cervical cancer-derived HeLa cells and observed the localization patterns of each protein before and after oxidative stress. Based on the data obtained from this large-scale imaging analysis, we constructed a Localizatome database, which will enable researchers to examine the localization of proteins under oxidative stress conditions and use this information for their biological studies.

## Materials and methods

### Cell culture and transfection

HeLa cells were cultured at 37°C and 5% CO_2_ in Dulbecco’s modified Eagle’s medium (Sigma-Aldrich) containing 10% fetal bovine serum (Gibco) and 1% penicillin/streptomycin (FUJIFILM Wako Pure Chemical Corporation). The cells were seeded into glass-bottom 96-well plates (Eppendorf) and transiently transfected individually with 10 287 fluorescent protein-fused human protein expression constructs (each 100 ng) using FugeneHD (Promega) according to the manufacturer’s instructions. No specific controls for transfection efficiency were included in this study.

### Fluorescent protein fusion human gene library

We used a yellow fluorescent protein (YFP) or a YFP variant Venus fusion human gene library developed by the National Institute of Advanced Industrial Science and Technology (AIST) [2], in which a fluorescent protein was fused at the C-terminus of the human full-length cDNA clones. The open reading frame (ORF) regions in each of the clones were derived from several libraries [12–18]. For genes with multiple splice variants, we chose the longest variant because it typically contains more motifs and domains, which is advantageous for genome-wide analysis of protein features, even when selecting the most abundant isoform in HeLa cells would be best. We finally selected 10 287 clones from a total of 17 065. The ORF information for each clone can be accessed through links to the Human Gene and Protein Database (HGPD, https://hgpd.lifesciencedb.jp/) in the Localizatome database.

### Fluorescence microscopy and image acquisition

Live-cell fluorescence imaging was performed using a high-content screening microscope (FDSS®/IMACS, Hamamatsu Photonics) customized with an ORCA-FLASH4.0 camera, a plate transport robot, and a dispenser. This microscope system was connected to an incubator (STR240, Liconic) via a customized plate transport system (MICRONIX), enabling the automated visualization of up to 200 96-well plates.

Using a 40× objective lens, cells seeded into glass-bottom 96-well plates were first observed at 20 h after gene transfection and then treated with arsenic trioxide (ATO) at a final concentration of 50 µM for 1 h to induce oxidative stress. For each gene (*n* = 1), Z-stack images (five slices with 1 µm intervals) of the same cells were captured in nine fields both before and after ATO treatment.

### Immunocytochemistry

Cells were washed with phosphate-buffered saline (PBS), fixed with 4% paraformaldehyde (FUJIFILM Wako Pure Chemical Corporation) for 10 min at 22–25°C and rewashed with PBS containing 0.05% TritonX-100. Cells were immersed in BlockAid (Thermo Fisher Scientific) for 60 min before incubating with primary antibodies overnight. The samples were then washed with PBS and incubated with secondary antibodies for 1 h before visualization. Primary antibodies used included anti-G3BP1 (13 057-2-AP; Proteintech) and anti-TIAR antibody (8509; Cell Signaling) to detect the stress granules. The secondary antibody used was Alexa Fluor 594 anti-rabbit IgG (A21207; Life Technologies). Hoechst (H3570; Life technologies) was used for nuclear staining.

### Image analysis and database construction

The acquired images were processed using the stack arithmetic program within the Methamorph software (Molecular Devices). For accurate focal plane selection, the images with the highest contrast from each Z-stack field were selected using an established auto-focusing approach. A machine learning program capable of detecting protein accumulation (Foci) was used to detect cells and score protein accumulation before and after stimulation [19]. Cells showing extremely high YFP- or Venus-tagged protein expression levels (maximum and mean cellular fluorescence intensity >50 000 and >2300, respectively) were excluded from analysis to avoid potential artifacts. Then, the change in accumulation scores before and after oxidative stress was calculated and saved as a CSV file along with the cell coordinate information. Image data and CSV files were uploaded to the Localizatome server. For database visualization, five cells showing significant changes in accumulation score were selected as representative examples. These representative cells were manually verified for protein accumulation changes during oxidative stress and displayed on the website along with the complete dataset. Nonetheless, all captured field images are available for download to enable independent analysis by users.

### Gene Ontology analysis and motif discovery

Gene Ontology analysis was performed using ShinyGO 0.80 [20]. For protein motif discovery, the STREME algorithm (an accurate and versatile sequence motif discovery tool) was used to identify 8–15 amino acid sequences [21]. Using this process, a background input was created from a list of 6140 human proteins that did not show oxidative stress-dependent accumulation (Foci Unchanged proteins). In addition, a disorder prediction score for each amino acid was calculated using IUPred3 [22].

## Results

### Protein localization analysis and database construction under oxidative stress

**Figure 1.**
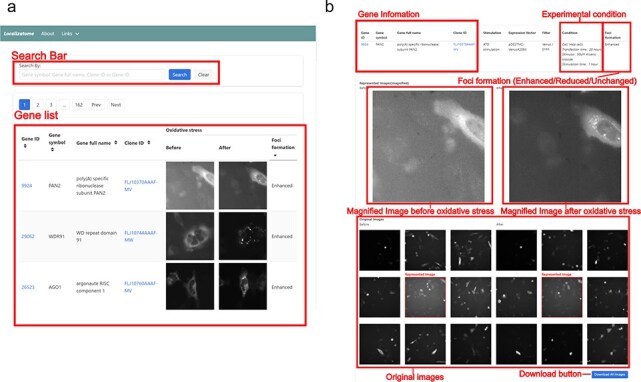
Screenshot of the Localizatome database. (a) The top page displays a search box and a list of images where users can search for genes using the gene ID, gene symbol, gene full name, or clone ID (FLJ ID), with gene IDs are linked to the NCBI database (https://www.ncbi.nlm.nih.gov/) and clone IDs are linked to HGPD (https://hgpd.lifesciencedb.jp/). (b) The individual gene page presents gene information and experimental conditions at the top, shows magnified images of representative cells with specific protein localization to investigate whether protein accumulation (foci formation) occurs in response to oxidative stress in the center, and provides original downloadable captured images at the bottom.

We used a human gene library containing YFP- or Venus-tagged proteins at the C-terminus to comprehensively analyse protein localization during oxidative stress. Before the main screening, we conducted control experiments to validate our experimental system. We confirmed that the Venus protein alone did not aggregate upon ATO treatment (Supplemental Fig. 1A) and optimized the transfection conditions (plasmid amount, transfection duration, and ATO treatment time) by ensuring that G3BP-Venus, a known stress granule marker, perfectly colocalized with endogenous G3BP1- and TIAR-positive stress granules (Supplemental Fig. 1B). Using these validated conditions, we transfected 10 287 plasmids individually into HeLa cells and conducted a large-scale imaging analysis using a customized high-throughput fluorescence microscopy system. This system systematically and objectively acquires data on the changes in protein subcellular localization. Other advantages include the ability to noninvasively and quantitatively observe protein dynamics in real-time before and after stress within the same cells. Furthermore, it provides subcellular localization information independent of antibody specificity and can deliver localization data even for proteins with low expression. To analyse stress-induced protein dynamics, we captured images of the same cells before and after ATO treatment, which induces oxidative stress. The resulting images were analysed using a machine learning program capable of detecting protein accumulation (Foci), detecting cells, scoring the accumulation of proteins within each cell, and calculating the change in scores before and after oxidative stress [19]. Protein localization changes were classified into three categories (Enhanced, Reduced, or Unchanged) based on both machine learning analysis and manual verification. The image data, cell coordinate information, and accumulation scores and changes were stored in the Localizatome database. This database was designed to be accessible through a user-friendly web interface and currently provides reliable data on the localization of 8055 proteins out of 10 287 observed proteins (Fig. 1A and B). To ensure data transparency and reliability, all original images can be viewed and downloaded directly from their corresponding gene pages. In addition, for validation purposes, each Clone ID is linked to the HGPD (https://hgpd.lifesciencedb.jp/), which provides detailed ORF information and steady-state localization data.

### Identification of oxidative stress-sensitive proteins

**Figure 2.**
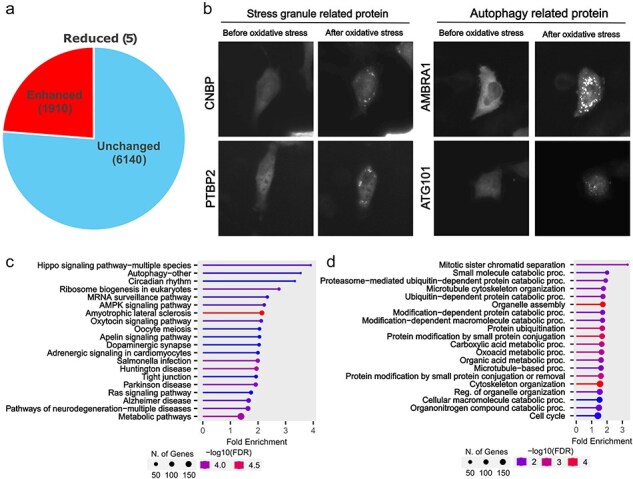
Proteins showing oxidative stress-dependent changes in localization. (a) In the Localizatome database, we identified 1910 proteins showing enhanced foci formation under oxidative stress. (b) Representative localization changes of stress granules and autophagy-related proteins. (c) Results of KEGG signal pathway analysis for the 1910 proteins that exhibited enhanced foci formation. (d) Results of Gene Ontology analysis (biological process) for the 1910 proteins that showed enhanced foci formation.

Although proteins were classified into three categories (Enhanced, Reduced, or Unchanged) based on their localization changes, we focused on comparing proteins showing Enhanced foci formation with those that remained Unchanged to identify proteins that actively respond to oxidative stress. From the 8055 proteins analysed by our machine learning program, we manually verified foci formation patterns and identified 1910 proteins showing enhanced foci formation under oxidative stress (Fig. 2A and Supplemental Table 1). These proteins included those already known to change their localization during oxidative stress, such as stress granules and autophagy-related proteins (Fig. 2B). Further analysis of these 1910 proteins by KEGG pathway analysis and Gene Ontology revealed that localization changes and foci formation of proteins related to the Hippo signalling pathway, cell division, and protein degradation. This finding is particularly relevant because oxidative stress inhibits cell division [23]. The phenotype of these accumulated proteins may represent one form of response to cell cycle arrest.

### Characterization of oxidative stress-sensitive proteins

**Figure 3.**
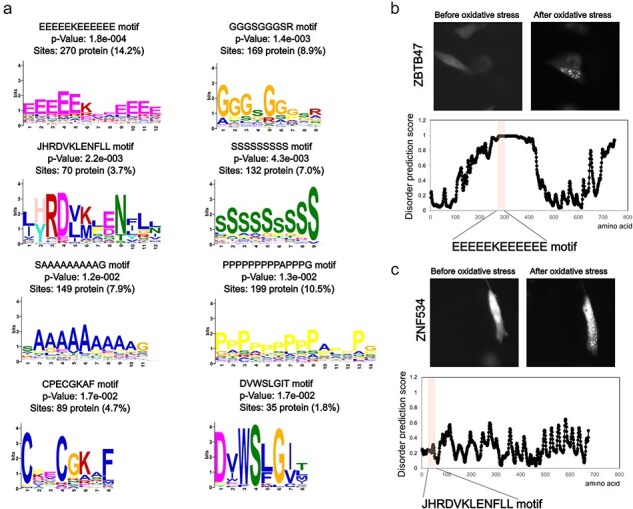
Sequence features of oxidative stress-dependent localization change proteins. (a) Motif analysis of proteins that show foci formation dependent on oxidative stress. (b) Representative localization changes of proteins with glutamic acid repeat motifs along with the disordered prediction score for each amino acid. (c) Representative localization changes of proteins containing the JHRDVKLENFLL motif along with the disordered prediction scores for each amino acid.

Next, we examined the sequence characteristics of these foci-enhanced proteins. Common motifs were searched based on the amino acid sequence information of this subset (Fig. 3A). Repeats of specific amino acids, such as alanine, glycine, proline, serine, and glutamic acid, or unknown motifs, such as JHRDVKLENFLL, may be involved in accumulation. In particular, the repetition of specific amino acid sequences is a characteristic of intrinsically disordered regions (IDRs). Moreover, proteins that showed enhanced foci formation in an oxidative stress-dependent manner showed high disordered prediction scores for repeated motifs (Fig. 3B). In the analysis of disordered regions, only amino acid content with a disordered prediction score >0.7 showed significantly higher values in the foci-enhanced protein group than in proteins with unchanged foci formation pattern (Supplemental Fig. 2). Recent studies on protein accumulation suggest that regions known as IDRs undergo liquid–liquid phase separation for certain protein groups [24–26]. Our results support the finding that the IDRs of proteins are involved in oxidative stress-induced accumulation. Motifs, such as JHRDVKLENFLL, CPECGKAF, and DVWSLGIT, did not contain typical IDRs based on the prediction tools (Fig. 3C). Further functional analyses of these motifs and the accumulation mechanism of proteins with these motifs are needed; however, information extracted from the Localizatome database may provide clues to unknown biological phenomena.

### Future directions

The Localizatome database is regularly updated and image data added. As our current analysis focusing on foci formation represents just one aspect of protein dynamics under oxidative stress, we plan to expand our analysis to include other parameters such as changes in expression levels, nuclear/cytoplasmic ratios, and membrane association patterns. Using our microscopy system and machine learning programs, we also aim to systematically compile databases on the intracellular dynamics of proteins involved in other important biological processes, such as cell differentiation, starvation, and endoplasmic reticulum stress. Updates and other information are described in the Localizatome database.

### Limitations

Although this database provides valuable information on stress-dependent changes in protein localization, several limitations should be noted. The effects of YFP/Venus fusion or missing localization signal sequences in the ORFs of our expression library may have resulted in the differing localization patterns of some proteins compared to their endogenous counterparts. Users can verify the ORF information through links to the HGPD provided for each clone ID. As previously reported, fluorescent protein fusion may affect protein localization, which should be carefully considered when interpreting the present data [27, 28]. Moreover, although we implemented measures to exclude cells with extremely high expression levels, we cannot completely rule out the effects of overexpression on foci formation. It is also important to note that our machine learning analysis and manual assessment results should be considered as reference data for genome-wide screening, rather than definitive determinations. The classification of foci formation into three categories (Enhanced/Reduced/Unchanged) may not fully capture the complexity of protein localization changes under stress conditions. To address these limitations, we made all microscopy images available for download, allowing independent analysis of our data.

## Conclusion

The Localizatome contains a dataset produced by combining a fluorescence protein fusion human gene library, a high-throughput microscopy system, and a machine learning program analysis to analyse changes in protein localization during oxidative stress. The Localizatome database represents a valuable tool for stress-related studies, providing both steady-state subcellular localization information and stress-dependent changes, which may lead to the discovery of new protein domains involved in protein accumulation. Furthermore, by integrating information from existing gene expression and proteomics databases with Localizatome data, researchers can explore novel functional protein domains and new cell structures.

## Supplementary Material

baaf028_Supp

## Data Availability

Localizatome is freely available at https://localizatome.embrys.jp/. All microscopy images can be downloaded through the database, although users should note that these are downsized versions optimized for web browsing. Original high-resolution raw data are available upon reasonable request.
