# Supplementary material for: Localizatome: a database for stress-dependent subcellular localization changes in proteins
Source: Database (Oxford). 2025 Apr 21;2025:baaf028. doi: 10.1093/database/baaf028 (PMC12010962; doi:10.1093/database/baaf028)
Supplement: baaf028_Supp [file baaf028_supp.zip › suppl_data/SupplementaryFigure_revise.pdf]

Localizatome: Database for Stress-Dependent Subcellular Localization Changes in Proteins

Takahide Matsushima, Yuki Naito, Tomoki Chiba, Ryota Kurimoto, Itano Keiko, Koji Ochiai, Koichi Takahashi, Naoki Goshima, Hiroshi Asahara

Supplemental Figure

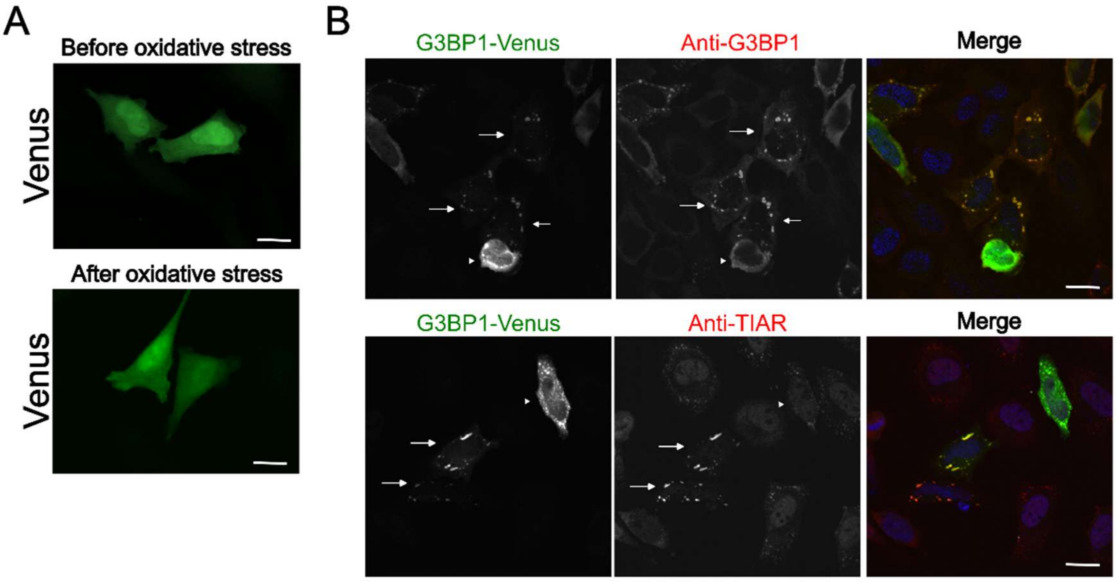

Supplemental figure 1. Optimization of screening conditions. (A) Aggregation properties of Venus fluorescent protein under oxidative stress. Analysis using HeLa cells expressing Venus fluorescent protein showed no aggregation under oxidative stress conditions. (B) Localization of G3BP1-Venus to stress granules. HeLa cells expressing G3BP1-Venus were analyzed by immunostaining with anti-G3BP1 and anti-TIAR antibodies. Arrows indicate cells where G3BP1-Venus perfectly colocalized with stress granules, while arrowheads indicate cells with high G3BP1-Venus expression showing impaired stress granule formation. Based on these imaging data, we excluded cells showing extremely high YFP/Venus-fusion protein expression (maximum fluorescence intensity >50,000 and mean cellular fluorescence intensity >2,300) from the screening analysis, as these cells showed ectopic localization changes and reduced the accuracy of our machine learning program. Scale bars show 20μm.

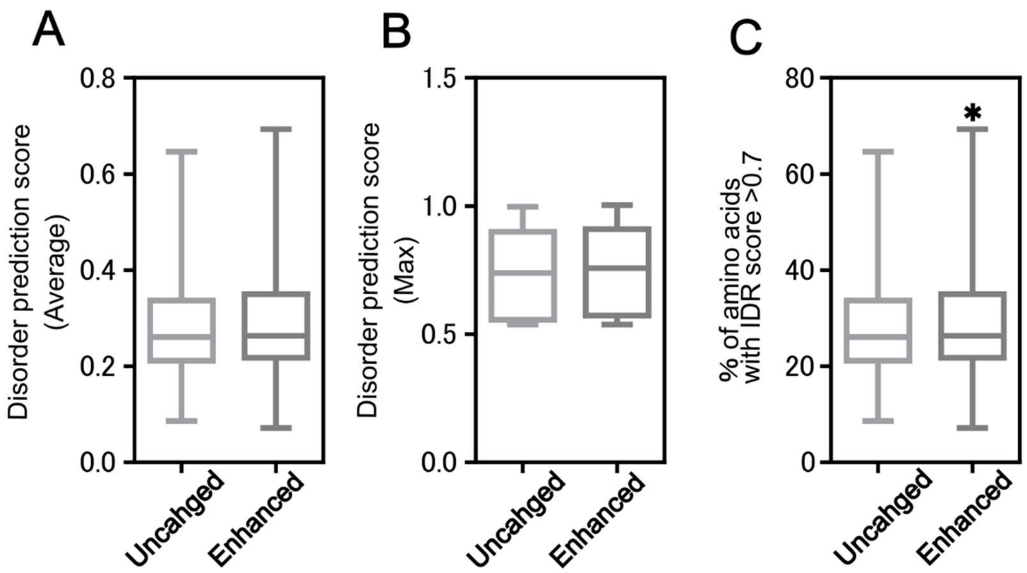

Supplemental figure 2. Intrinsic Disorder Regions (IDR) of Proteins Undergoing Oxidative Stress-Dependent Localization Changes. Using disordered prediction tools, the average (A), maximum (B), and amino acid content with a disordered prediction score above 0.7 (C) were calculated for proteins with [Enhanced, 1910 proteins] and without [Unchanged, 6140 proteins] foci formation. An asterisk represents statistical significance ($p < 0.05$), as assessed by Student's t-test.
